# Germline mutation landscape of Chinese patients with familial breast/ovarian cancer in a panel of 22 susceptibility genes

**DOI:** 10.1002/cam4.2093

**Published:** 2019-04-13

**Authors:** Jiayu Wang, Weiwei Li, Yujian Shi, Yan Huang, Tao Sun, Lili Tang, Qing Lu, Qiumo Lei, Ning Liao, Feng Jin, Hui Li, Tao Huang, Jun Qian, Danmei Pang, Shusen Wang, Peizhi Fan, Xinhong Wu, Ying Lin, Haiyan Qin, Binghe Xu

**Affiliations:** ^1^ State Key Laboratory of Molecular Oncology, Department of Medical Oncology National Cancer Center/Cancer Hospital Chinese Academy of Medical Sciences & Peking Union Medical College Beijing China; ^2^ Top Gene Tech (Guangzhou) Co., Ltd. Guangzhou China; ^3^ Department of Breast Surgery Chinese People's Liberation Army Beijing China; ^4^ Department of Medical Oncology Liaoning Cancer Hospital Shenyang China; ^5^ Department of Breast Surgery Hunan Cancer Hospital Changsha China; ^6^ Department of Breast Surgery West China Hospital of Sichuan university Chengdu China; ^7^ Department of Breast The Third Hospital of Nanchang Nanchang China; ^8^ Department of Breast Guangdong General Hospital Guangzhou China; ^9^ Department of Breast Surgery The First Hospital of China Medical university Shenyang China; ^10^ Department of Breast Surgery SiChuan Cancer Hospital Chengdu Sichuan China; ^11^ Department of Breast Surgery Union Hospital of Tongji Medical College, Huazhong University of Science and Technology Wuhan China; ^12^ Department of Breast Surgery The First affiliated Hospital of bengbu medical college Benghu China; ^13^ Department of breast cancer oncology Foshan Hospital of Sun Yat‐Sen Unversity Foshan China; ^14^ Sun Yat‐sen University Guangzhou China; ^15^ Department of Breast Xiangya Hospital of Central South University Changsha China; ^16^ Department of Breast Hubei Cancer Hospital Benghu China; ^17^ Department of Breast the First affiliated Hospital of Sun Yat‐Sen Unversity Guangzhou China

**Keywords:** *BRCA1*, *BRCA2*, familial breast cancer, multigenes, novel mutation

## Abstract

Genetic testing for germline mutations in *BRCA1/2* of patients with breast cancer (BC) is part of routine patient care. However, *BRCA1/2* mutations account only for a fraction of familial BC. A custom panel of 22 gene sequencing was performed on each patient. Among the 481 female patients, 135 patients were detected to carry pathogenic (P)/likely pathogenic (LP) mutations (28.1%), which corresponded to 12 different cancer predisposition genes [14.6% (70/481) on *BRCA1* gene, 5.0% (24/481) on *BRCA2 *gene, 8.5% (41/481) on non‐*BRCA1/2* genes]. Moreover, 24.7% (119/481) of patients had mutation of unknown significance (VUS) in these genes. The most common (8/481) pathogenic mutation is *BRCA1* c.5470_5477del, while *BRIP1* 2392 C > T of patients was detected. All the mutations detected were mainly seen in the homologous recombinant repair pathway. Compared to *BRCA2* mutation, *BRCA1 *mutation is higher in younger female patients (*P* < 0.01). Some pathogenic mutations were detected in the patients’ familiy members without the past history of tumor and 92 novel mutations were detected (31 on BRCA including 2 P, 16 LP, 13 VUS; 61 on non‐*BRCA1/2 *including 9 LP, 52 VUS). The detection rate of *BRCA1/2* mutations was higher in patients with three or more cancer family members than those with one or two. However, the difference was not statistically different. The results suggest that multigene panel testing can increase mutation detection rate for high‐risk BC patients. Detailed family history can help to categorize new mutations.

## 1 INTRODUCTION

*BRCA1/2* mutations are characterized as an increased lifetime risk for hereditary breast and ovarian cancer syndrome.^1^ Clinical genetic testing for familial breast cancer (BC) has been transformed by the advent of massively parallel sequencing, which allows simultaneous screening of a large number of genes at a fraction of the cost on one gene sequencing previously.^2^ However, there is a large portion of familial BC not associated with *BRCA1/2* mutations. Familial BC often related to mutations of non‐*BRCA1/2* genes in homologous recombination (HR) pathway (*ATM*, *CHEK2*, *BARD1*, *BRIP1*, *MRE11A*, *NBN* and etc), by DNA damage response pathway (*MSH2*, *MLH1*, *MSH6*, *PMS2 *and etc) 3, 4 and mismatch recognition pathway (*MUTYH*, *EPCAM* and etc).^5^, ^6^ Mutations of these genes have been reported to have medium‐to‐high penetrance of hereditary BC.^7^, ^8^, ^9^ The prevalence and spectrum of BC germline mutations in Chinese female patients have not been well investigated. Meanwhile, they are important for patient management.

In this study, we used a custom‐designed 22‐gene panel (Table 1) in order to to evaluate the clinical value of multigene panel testing in Chinese patients with familial BC. Most of the genes are associated with hereditary BC.

**Table 1:** Clinicopathological characteristics between mutation carriers and noncarriers in 481 patients

Characteristics	Noncarriers (N = 227)		*BRCA1* carriers (N = 70)		*BRCA2 *carriers (N = 24)		Others (N = 41)		*P1*	*P2*	*P3*
	No.	%	No.	%	No.	%	No.	%			
Age at diagnosis, years											
Mean ± SD	47.5 ± 10.6	43.2 ± 10.2							<0.01	0.991	0.011
≤40 years	40	17.60%	31	44.30	5	20.80	15	36.60			
>40 years	168	74.00%	33	47.10	18	75.00	23	56.10			
Unknown	19	8.40%	6	8.60	1	4.20	3	7.30			
Family history of other cancer									<0.01	0.955	0.998
Yes	41	18.10%	29	41.40	5	20.80	8	19.50			
No	186	81.90%	41	58.60	19	79.20	33	80.50			
Unknown	0	0.00%	0	0.00	1	4.20	0	0.00			
Lateral of breast cancer									0.068	1	0.596
Bilateral	6	2.60%	6	8.60	1	4.20	0	0.00			
Unilateral	175	77.10%	51	72.90	20	83.30	35	85.40			
Unknown	46	20.30%	13	18.60	3	12.50	6	14.60			
Class									<0.01	<0.01	0.868
TNBC	12	5.30%	27	38.60	19	79.20	2	4.90			
Non‐TNBC	132	58.10%	24	34.30	2	8.30	33	80.50			
Unknown	23	10.10%	19	27.10	3	12.50	6	14.60			
History									0.081	0.075	0.135
Ductal	86	37.90%	35	50.00	14	58.30	21	51.20			
Others	141	62.10%	35	50.00	10	41.70	20	48.80			

*P1*: *BRCA1* carriers vs noncarriers; *P2*: *BRCA2 *carriers vs noncarriers; *P3*: Others non‐*BRCA1/2* genes carriers vs noncarriers

## 2 MATERIALS AND METHODS

### 2.1 Study cohort

Total of 481 female BC patients were selected in 28 hospitals in China from 2016 to 2017. Patients whose first and secondary degree family members diagnosed of breast or ovarian cancer were included in this study. Primary BC patients with family history were selected by the attending doctors, or they volunteer to participate. All the patients signed the informed consent.

### 2.2 Multigene panel design

In this study, 22 cancer susceptibility genes (Table S1) were included in this panel for their possible role in the development of hereditary cancer based on published literatures. All exons, partial intronic and UTR regions of these genes were covered by this panel which consists of 120 kb pairs approximately. Probes of this panel were synthesized by iGeneTech (China).

### 2.3 Next‐generation sequencing and data processing

Genomic DNA (gDNA) was extracted from peripheral blood samples (2‐5 ml) of each patient using QIAamp DNA Blood Midi Kit (Qiagen, Germany) according to manufacturer's instruction. The target gene library was generated using KAPA Hyper prep Kits (Roche NimblGen, INC). The prepared libraries were sequenced by NextSeqCN500 (BerryGenomics, China). The sequencing depth was about 1000X. Qualified reads were aligned to human reference genome hg19 by Burrows‐Wheeler Alignment (BWA 0.5.9). Germline mutations were detected using Genome Analysis Toolkit (GATK) and SAMtools. Annotations were defined using ANNOVAR (http://www.openbioinformatics.org/annovar). Population allele frequencies were extracted from ExAC (http://exac.broadinstitute.org/), GnomAD (http://gnomad.broadinstitute.org/) and 1000 Genomes Project (http://www.1000genomes.org). Mutation databases including HGMD (http://www.hgmd.cf.ac.uk/), OMIM (http://omim.org/), ClinVar (http://www.ncbi.nlm. nih.gov/clinvar/), and BIC (https://research.nhgri.nih.gov/bic/) were also included in the analysis pipeline. In this study, we just analyzed point mutations, short insertion, and deletions. In addition, mutations (pathogenic, likely pathogenic, VUS) were confirmed by Sanger sequencing.

### 2.4 Germline mutation classification

All mutations were classified according to the American College of Medical Genetics (ACMG) professional practice and guidelines [five‐tier mutation: P (Pathogenic); LP (Likely Pathogenic); uncertain significance (VUS); LB (Likely Benign); and B (Benign)].^10^ Mutation classification was generated by genetic Counselor and verified by two curators.

### 2.5 Statistical analysis

Statistical analysis of clinicopathological characteristics between mutation carries and non‐carries were performed by the Chi‐square test or the Fisher exact test. Two‐sided *P* values less than 0.05 was considered to be statistically significant. All analyses were performed using R language (https://www.r-project.org/).

## 3 RESULTS

### 3.1 Mutation status and patient clinical characteristics

In this study, blood samples from 481 Chinese BC patients who have a family history were analyzed by using a custom panel of 22 genes. The median age at diagnosis was 47 years (range, 19‐77 years). The younger group (<40 years) carried more *BRCA1* pathogenic mutations than the elder group (>40 years) (17.6% vs 44.3%, *P* < 0.01) (Table 1). Moreover, the rate of non‐negative mutation in *BRCA1/2* detected (including P, LP, VUS) in the younger group (≤50 years) was higher than that of the elder group (>50 years) (25% vs 9.9%). The mutation rate in non‐*BRCA1/2* mutation carriers and non‐carriers are similar (Figure S1). Mutation rates of *BRCA1* (5.3% vs 38.6%, *P* < 0.01) and *BRCA2* (5.3% vs 79.2%, *P* < 0.01) were higher in the triple‐negative breast cancer (TNBC) group compared to non‐TNBC group (Table 1). A patient diagnosed with TNBC carried two pathogenic mutations c.2155A > T and c.2143_2147delCTGGT in *BRCA1 *gene. We found only one study that reported a similar case.^11^ Compared with noncarriers, the mutation rate of *BRCA1 *was higher in patients without a BC family history while the mutation rate of *BRCA2* and other genes showed no statistically significant difference in age of diagnosis, family history of other cancer, lateral of BC, and tumor type.

### 3.2 Rate and spectrum of germline mutations in female Chinese patients with familial BC

**Figure 1:**
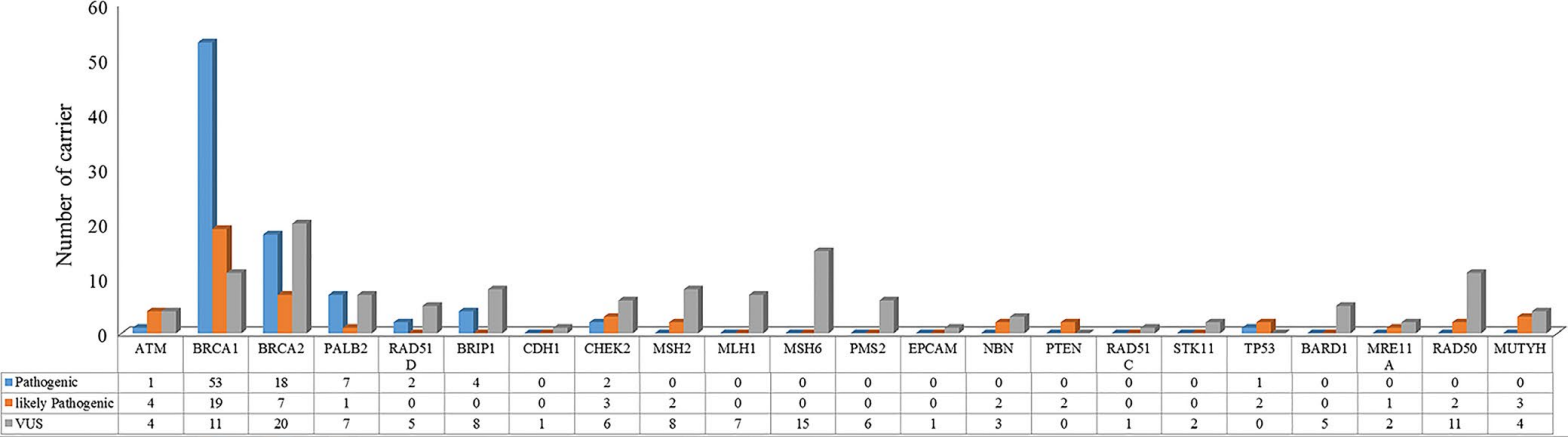
Distribution of different mutations identified with multiple‐gene panel

**Figure 2:**
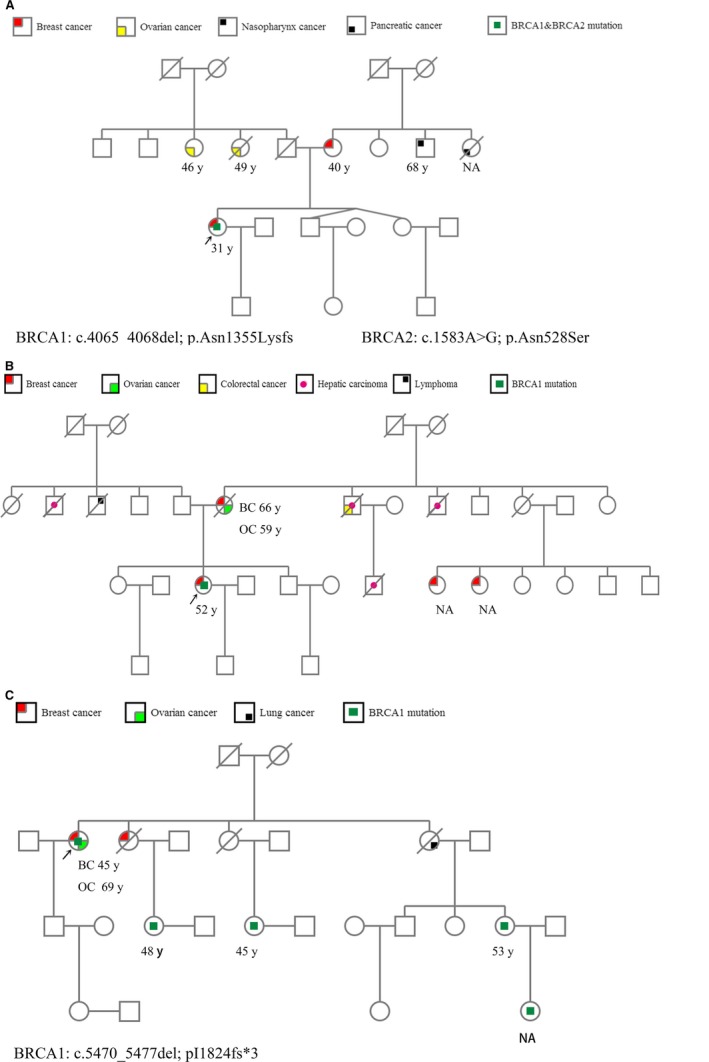
Pedigrees. (A) Pedigree of patient with c.4065_4068del; p.Asn1355LysfsTer mutation in *BRCA1 *gene. (B, C) Pedigree of patients with c.5470_5477del; p.I1824fs*3 mutation in *BRCA1* gene. The probands are indicated by arrowheads. Cancer type and age at cancer diagnosis are indicated in the legend

Sequencing results of the custom 22 gene panel showed that 135 (28.1%) of the 481 patients carried at least one pathogenic (LP and P) mutation and 70 (14.6%) and 24 (5.0%) patients carried pathogenic mutation of *BRCA1* and *BRCA2*, respectively. The remaining 41 (8.5%) patients carried mutations in non‐*BRCA1/2* genes (Figure 1 and Table S2). Furthermore, 39 (8.1%) patients carried more than one mutation. For example, one patient was identified to carry three mutations: *BRCA1* (c.5202del, P), *BRCA2* (c.4408_4412del, P), and *TP53* (c.884C > T, VUS) (Tables S3 and S4). Two *BRCA1* mutations were present in patients with a family history of breast, ovarian, pancreatic, and nasopharygeal cancer (Figure 2A). Among the mutations detected in this cohort, 73 of 104 (70.2%) pathogenic mutations were in *BRCA1/2* genes (50 in *BRCA1*, 23 in *BRCA2*), while 41 (29.8%) were in non‐*BRCA1/2* genes (Figure 1). The *BRIPI* c.2392C > T (n = 1) was detected in patients with a family history of nasopharyngeal carcinoma in addition to BC. The most frequent pathogenic mutation in this study is c.5470_5477del of *BRCA1* was identified in eight families (Table S5). As shown in Figure 2B, the family with this mutation has a apparent cancer family history. The mutation was found in one concurrent BC and ovarian cancer patient and her four healthy family members (Figure 2C). It is noteworthy that the patient’s daughter was not a carrier, while her second, and third degree female relatives (sister's daughter and sister’s granddaughter) were carriers. Moreover, thirteen mutations in BC‐related genes were detected in more than one family (Table S5).

**Figure 3:**
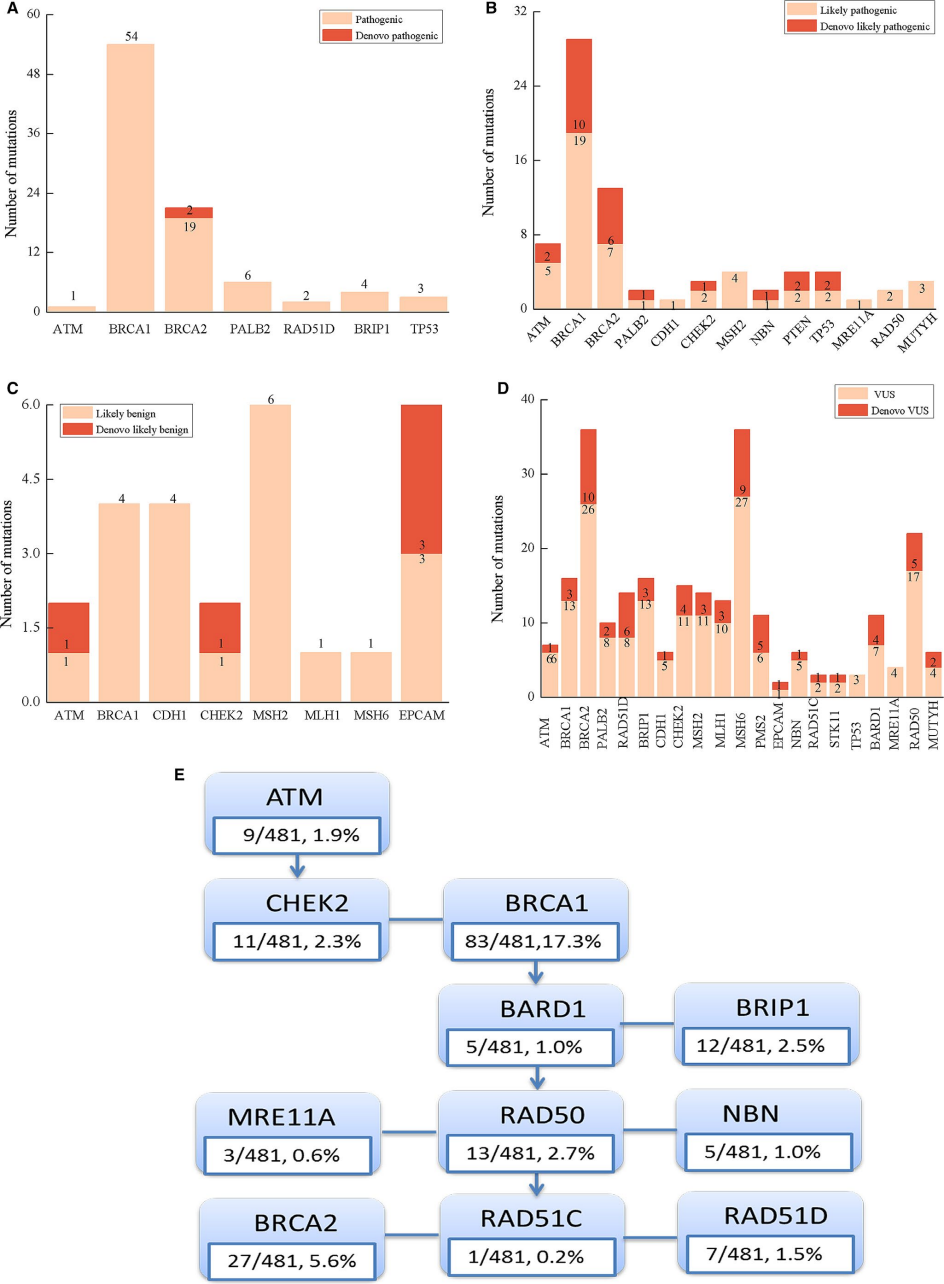
The reported mutations were identified by the multiple‐gene panel method. (A) (B) (C) (D) The number of novel and non‐novel mutations in non‐negative mutations (Pathogenic/likely Pathogenic/likely benign/unknown significance). (E) The percent of gene muations in homologous recombination pathway

More VUSs were found in non‐*BRCA1/2* than *BRCA1/2* genes. Here, 42 (27.5%) of 153 patients carried VUS in *BRCA1/2* genes while 127 in non‐*BRCA1/2* genes (16 patients carried both *BRCA1/2* and non‐*BRCA* mutations) (Figure 1). It was found that at least one VUS was identified in *EPCAM*, *MLH1*, *MSH6*, *PMS2*, *RAD50*, *RAD51C*, *STK11*, and *BARD1* genes, respectively. The mutaion type of VUSs is mainly SNVs, which were found in more than one patient with significant family history. For example *MSH6 *c.3244C > T was found in 3 families (Table S2). In addition, 31 mutations detected in this cohort in *BRCA1/2 *genes and 61 mutations in non‐*BRCA1/2* genes were not found in the BIC database (Figure 3A‐D)*.* The discussion of novel mutations was based on the disease onset age, cancer type, unilateral/bilateral lesion, and family history.^10^ Meanwhile, the type of mutation is important supporting evidence for the classification of novel mutations. The *BRCA1* c.3919G > T is a nonsense mutation, which was detected in two families with a family history of BC from different regions in China.

The pathway enrichment study further confirms that a majority of mutations (39.7%; 191/481) were identified in the HR (Figure 3E). In the MMR‐related genes, only one likely pathogenic mutation was detected in the *MSH2* gene (c.2197G > A, 4 family), which was found in patients diagnosed with pediatric medulloblastoma.^12^

**Figure 4:**
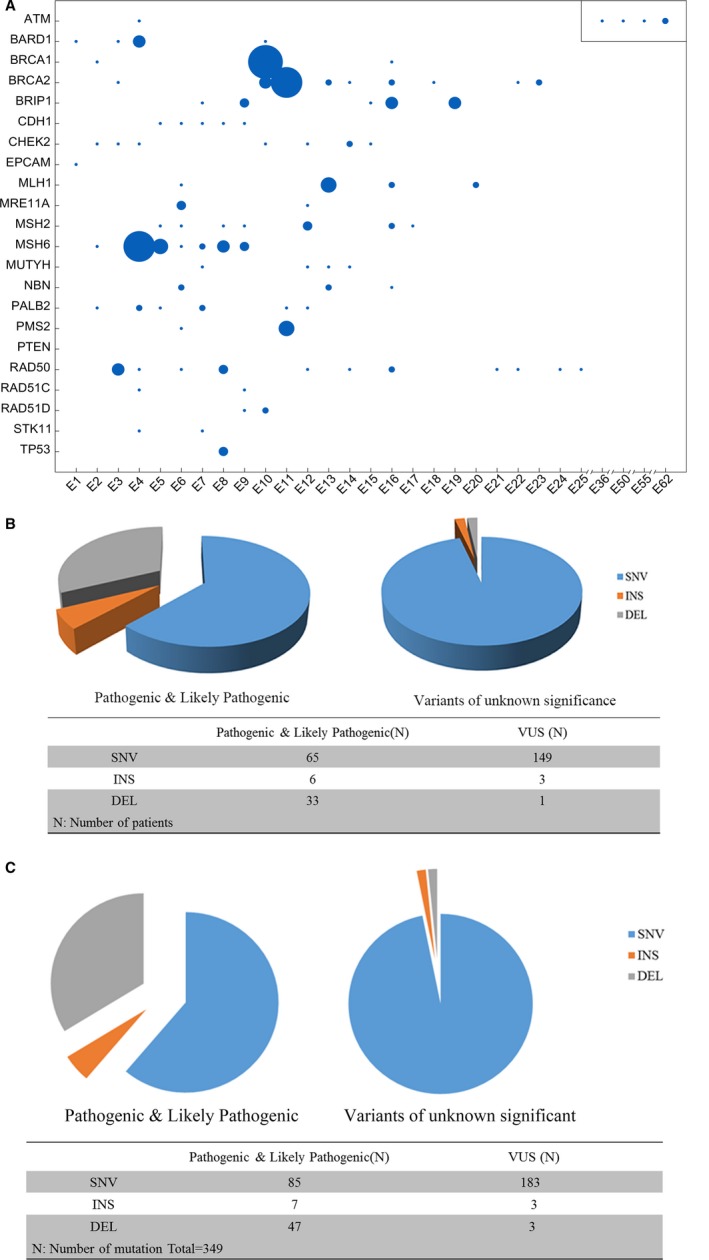
The distribution of germline mutations in BC families. (A) The distribution of 22 gene mutations in exon regions. (B) Distribution of different mutations identified with multiple‐gene panel

In this study, we found that sites of the mutation were scattered in different regions of a gene (Figure 4A). Here, 257 patients were detected with gene mutations (104/257 P, 50/257 LP, 151/257 VUS) in this study, including SNVs (214), insertions (Ins) (9) and deletions (Del) (34) (Figure 4B). The distribution of mutations is close to that in the whole population (Figure 4C).

**Figure 5:**
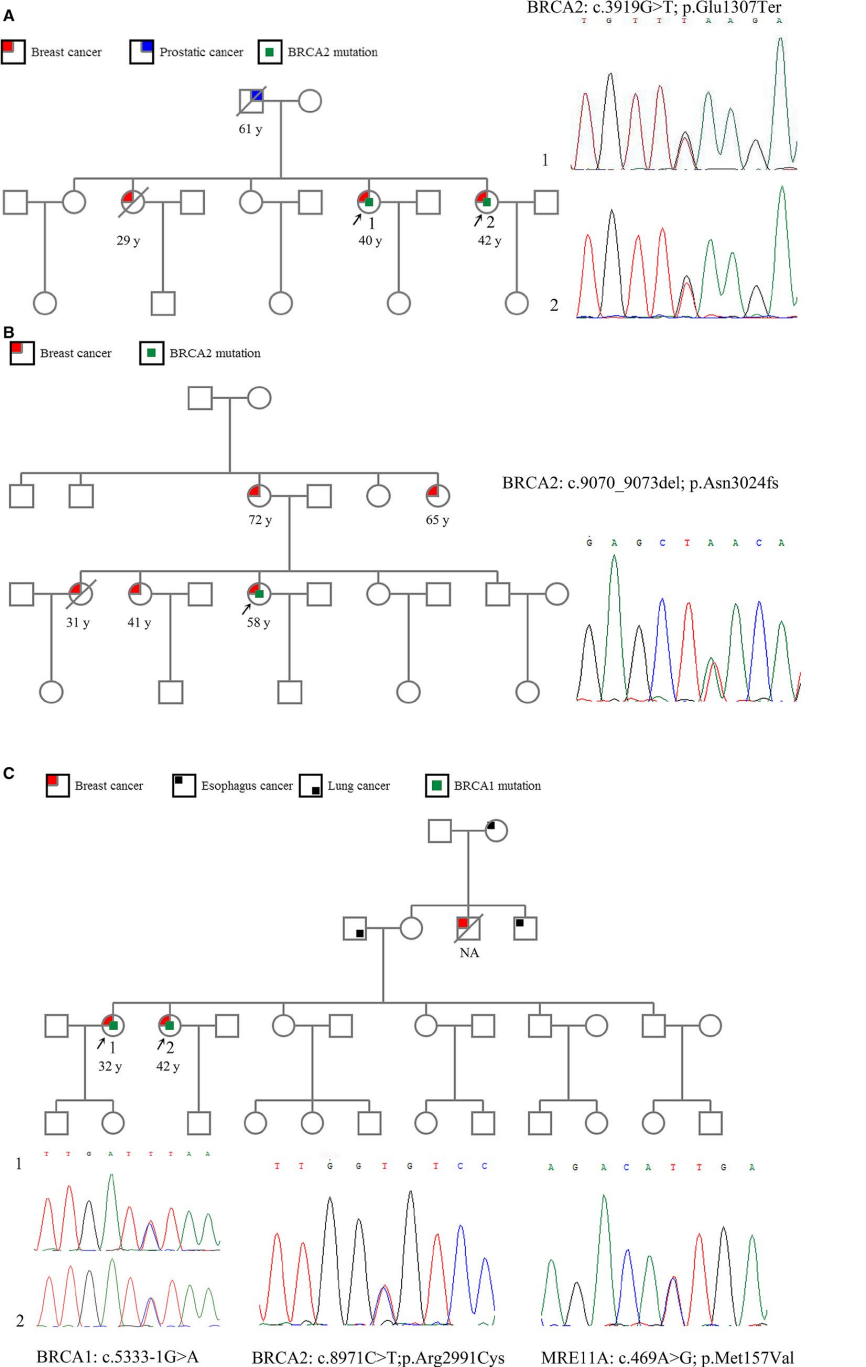
Pedigrees. (A) Pedigree of patient with c.3919G > T; p.Glu1370Ter mutation in *BRCA2 *gene. (B) Pedigree of patient with c.9070_5073del; p.I1824fs*3 mutation in *BRCA2* gene. (C) Pedigree of patient with three different mutations in *BRCA1*, *BRCA2* , and *MRE11A* genes. The probands are indicated by arrowheads. Cancer type and age at cancer diagnosis are indicated in the legend

Family history studies demonstrated that BC is the most common type (230/481, 47.8%) followed by ovarian (37/481), colorectal (21/481), gastric (20/481), and cervical (12/481). Twenty‐two patients (22/481) had a history of more than one cancer type (Table S6). The pathogenic *BRCA2* (c.3919G > T) mutation was detected in one family with a history of BC and prostatic cancer (Figure 5A). The patient with the *BRCA2* (c.9070_9073del) mutation had a family history of four BC relatives (Figure 5B). In addition*,* the family with lung and esophageal cancers, two VUS *BRCA2* and *MRE11A* mutations were detected in two BC patients with *BRCA1* likely pathogenic mutation (Figure 5C). In eight families, even though there were three relatives in each family had history of cancer, there were no mutations detected by the panel (Table S4). In general, there was no significant difference between gene mutation and family history. However, the data showed that there is a linear trend, which means that more relatives with cancer, the higher proportion of probands with *BRCA* mutations (Figure S2).

## 4 DISSCUSSION

In our cohort study of 481 patients who underwent genetic testing, *BRCA1* mutations were significantly enriched in younger patients (<40 years). *BRCA1/2* mutations were observed more in the TNBC patients.^13^ All 22 genes had higher detection rates in patients less than 50. Thus, we concluded that genetic screening in this populations is essential.^14^

In this study, pathogenic mutations were identified in 135 patients most of them in the *BRCA1/2* gene.The mutation carrier rate was higher than the unselected BC patients (19.5% vs 5.3%).^15^ Mutations found in the *BRCA1* gene (14.6%) in this cohort were almost three times higher compared to *BRCA2* (5%). A higher mutation burden in *BRCA1* was also reported in other studies in patients with a cancer history or unselected patients in Asian population.^16^, ^17^ Besides, 8.5% of patients carried non‐*BRCA1/2* pathogenic mutations, which were mainly found in *ATM*, *CHEK2*, *PALB2*, and *BRIP1* genes.^7^ Previous studies have reported that approximately 11.4% of BC patients carried mutations in non‐*BRCA1/2* genes.^8^ It is worthnoting that rare mutations of* BRIP1* c.2392C > T was detected in one patient in our cohort. However, this mutation is reported in three studies and found in 18 Fanconi anemia patients, and is associated with BC in the Irish.^18^, ^19^ Multigene panel testing is likely to provide a more complete mutation capture than *BRCA1/2* alone.^9^, ^20^ No pathogenic mutations were found in *STK11* and *RAD51C*. It was also reported that *STK11 *mutations have been limited to individuals with clinical features indicative of the Peutz‐Jegher syndrome and *RAD51C* mutations reported in OC.^17^ Of note, *BARD1 *and *BRIP1* pathogenic mutations were not detected in this study . Other studies showed that *BARD1 *mutation might be rare and responsible for a few familial BC patients.^14^ It has been reported that *BRIP1* had a higher penetrance for OC.^21^ On the other hand, about 47.2% (227/481) patients had no pathogenic mutations detection in our cohort. One possible explanation for this was that only 22 genes was included in the panel and there are other genes involved in BC patients.^7^, ^8^ Furthermore, the limitation of the detection method used may affect mutation detection.^20^

About 24.7% (119/481) of patients carried VUS mutations, mostly in non‐*BRCA* genes.^2^ Previous studies often focused on *BRCA* genes other than non‐*BRCA* genes, classification of VUS was investigated in more detail and some of them were classified as non VUS.^10^ The rate of VUS detection depends on the number of genes included and has been reported ranging from 6.7 (6‐gene panel) to 41.7% (25‐gene panel).^8^, ^22^ In this study, the rate of VUS was 24.7%, partialy for the study included patients with a family history of multiple tumors. Some of the VUS will eventually be classified as non‐VUS with further investigations. However, segregation analysis in the same family is not conducive to the categorization of the VUS. The multifactorial approach should be considered in this process.^23^ Throughout all the mutations, it is worthnoting that the pathogenic mutations are more frequent in nonsense and frameshift mutations while VUS mutations are more missense. This also confirmed that the mutation type had a large difference according to the structure and function of genes, among which nonsense and frameshift mutations were likely to be pathogenic.^10^ This study showed that a majority of mutations identified were in the HR signaling pathway.^21^ Therefore, this pathway might play an important role in familial BC in China.

Some patients with a unique family history were discussed in this study. The common characteristics of these families were with multiple cancers diagnosed and multiple family members affected. Most of the tumors were associated with hereditary BC, such as colorectal, endometrial, ovarian cancer, and pancreatic cancer, and so on. These results are consistent with previous findings that the increase in the incidence of pancreatic cancer was related with *BRCA *mutation.^12^ Phelan et al, suggested that CRC screening should be done among women with *BRCA1* mutation.^24^ The number of relatives with BC were positively correlated with *BRCA1/2* mutations. It is one of the characteristics of hereditary tumor. Family studies supported the selection of management choices, especially for carriers without cancer.^15^ Additionally, familial BC is not only related to colorectal and other cancers, but also to other rare cancer types that should be investigated further in some families. This study also indicated that selected population screening may help to reduce cancer‐related mortality.^23^

In conclusion, appropriately selected patients may benefit from multiple‐gene sequencing, especially those with personal or family history of more than one possible genetic syndrome. The detailed family history and clinical characteristics are useful for mutation classification, specifically in cases of unreported mutations. Moreover, family separation is more valuable in the classification of pathogenic/likely pathogenic mutations rather than VUS. Our findings are important for the promotion of large panels in high‐risk breast/Ovarian cancer populations and clinical genetic testing of patient management.

## CONFLICT OF INTEREST

The authors declare no conflict of interest.
